# Effect of Early Intravenous Immunoglobulin Therapy in Kawasaki Disease: A Systematic Review and Meta-Analysis

**DOI:** 10.3389/fped.2020.593435

**Published:** 2020-11-20

**Authors:** Fan Yan, Huayong Zhang, Ruihua Xiong, Xingfeng Cheng, Yang Chen, Furong Zhang

**Affiliations:** ^1^Intensive Care Unit, Wuhan Children's Hospital (Wuhan Maternal and Child Healthcare Hospital), Tongji Medical College, Huazhong University of Science & Technology, Wuhan, China; ^2^Department of Cardiology, Wuhan Children's Hospital (Wuhan Maternal and Child Healthcare Hospital), Tongji Medical College, Huazhong University of Science & Technology, Wuhan, China

**Keywords:** Kawasaki disease, intravenous immunoglobulin therapy, coronary artery lesion, coronary artery aneurysm, IVIG unresponsiveness

## Abstract

**Background:** In the latest 2017 American Heart Association guidelines for Kawasaki disease (KD), there are no recommendations regarding the early administration of intravenous immunoglobulin (IVIG). Therefore, the purpose of this systematic review and meta-analysis was to investigate the effects of early IVIG therapy on KD.

**Methods:** We searched databases including the PubMed, Medline, the Cochrane Library, and the Clinicaltrials.gov website until July 2019.

**Results:** Fourteen studies involving a total of 70,396 patients were included. Early treatment with IVIG can lead to an increased risk of IVIG unresponsiveness [OR 2.24; 95% CI (1.76, 2.84); *P* = 0.000]. In contrast to the studies performed in Japan [OR 1.27; 95% CI (0.98, 1.64); *P* = 0.074] that found no significant difference in coronary artery lesions (CAL) development, studies conducted in China [OR 0.73; 95% CI (0.66, 0.80); *P* = 0.000] and the United States [OR 0.50; 95% CI (0.38, 0.66); *P* = 0.000] showed a reduced risk in the occurrence of CAL with early IVIG treatment.

**Conclusions:** At present, the evidence does not support the treatment with IVIG in the early stage of the onset of KD. But, early IVIG treatment could be a protective factor against the development of CAL, which needs to be further clarified.

## Introduction

Kawasaki disease (KD) is an acute self-limiting disease leading to vasculitis that predominantly affects infants and young children. Coronary artery lesion (CAL) is the most common complication. In severe cases, giant coronary artery aneurysms or coronary artery ectasia can develop, which is the leading cause of acquired heart disease (1, 2). Intravenous immunoglobulin (IVIG) therapy is the first-line treatment of KD with well-established therapeutic effects in preventing coronary artery abnormalities (3). Coronary artery aneurysms (CAAs) develop in ~25% of untreated patients (4); however, in patients receiving a timely high dose of IVIG, it is reported that only about 5% of patients (5–7).

The mechanism as to why IVIG is effective in the treatment of KD is unknown. IVIG is considered to reduce the prevalence of CAL by regulating the immune system, including the modulation of cytokine production, neutralization of bacterial superantigens or other etiologic agents, and suppression of endothelial cell activation. The 2004 American Heart Association (AHA) guidelines of KD stated that IVIG was recommended to be used within 10 days after the onset of the disease and, if possible, within seven days. And, according to a study by Muta and Fong, early administration of IVIG (within 5 days) seemed to show no significant improvement in the cardiovascular outcomes, but there was an increased need for IVIG retreatment (4, 8, 9). In the latest AHA guidelines for KD in 2017, it is suggested that for experienced clinical experts, the diagnosis of KD can be made as early as 3 days after the onset of disease if typical symptoms are present; however, there is no guideline on the timing of IVIG administration and if it can be given earlier (10).

Therefore, in our research, studies meeting the inclusion criteria were included to compare the outcomes of KD children treated with IVIG at early and routine time, including coronary artery outcome and non-response to IVIG, so as to understand the effects of early IVIG therapy in KD and to provide evidence on the timing of IVIG administration.

## Methods

This systematic review and meta-analysis was reported in accordance with the Preferred Reporting Items for Systematic Reviews and Meta-Analyses (PRISMA) Statement (11) (Supplementary Checklist 1).

### Search Strategy

We searched databases including the PubMed, Medline, the Cochrane Library, and the Clinicaltrials. gov website until July 2019 without language restrictions. The keywords and subject terms were (“immunoglobulin” OR “IVIG” OR “immune globulin”) and (“Kawasaki disease” OR “Mucocutaneous Lymph Node Syndrome” OR “Kawasaki Syndrome”).

### Study Selection Criteria

The population, intervention, comparison, and outcome approach was used for the study inclusion. The population of interest were children diagnosed with KD. The intervention of interest was the treatment using IVIG as the initial therapy and studies had to include a comparison between the early treatment with IVIG (IVIG administration <5 days of onset of disease) and ≥5 days of KD onset. Outcome measurements included the incidence of CAL, IVIG unresponsiveness, and CAA.

Meanwhile, the studies included fulfilled the following criteria: (1) sufficient data to extract the number of cases and controls in each group or provision of odds ratios (ORs) with 95 % confidence intervals (CIs) and (2) the types of studies should be randomized controlled or non-randomized controlled clinical trials, case-control, cross-sectional, or cohort studies.

### Data Collection and Assessment of Study Quality

For each eligible study, the following information was independently extracted by two researchers (FY and HZ): last name of first author, study design, duration of follow-up, study duration, study location, age of participants, proportion of males, sample size in each group (subgroup by time of IVIG treatment), diagnostic criteria of CAL, definition of IVIG unresponsiveness KD, outcomes, and quality criteria. The quality of the included studies was also independently assessed by two researchers (FY and HZ) using the Newcastle-Ottawa scale (12) (Supplementary Tables 1, 2). When disagreements arose, group discussions would be carried out to resolve it.

### Outcome Measures

The primary outcomes were CAL and IVIG unresponsiveness. CAL included the coronary artery dilation, CAA, and coronary artery stenosis meeting the definition of the Japanese Ministry of Health criteria, coronary artery Z score system, or Chinese literature (13–15). IVIG unresponsiveness was defined as a persistent or recrudescent fever in a period after the completion of the first IVIG infusion. The secondary outcome was CAA.

### Statistical Analysis

We estimate the pooled odds ratio (OR) with a 95% confidence interval (CI) using study-specific ORs abstracted or calculated from raw data; results from a multivariate analysis or adjusted ORs were preferred to those of univariate analysis or crude results. Considering the within– and between–study variability, the random effects model was chosen. Heterogeneity among the studies was assessed using the *Q* test. It was quantified by *I*^2^ values and 25, 50, and 75% were considered as low, moderate, and high levels of heterogeneity, respectively. To test the publication bias, the Begg's and Egger's tests were used. *P* < 0.1 indicated that a publication bias existed. Sensitivity analysis was performed by evaluating the pooled estimate after omitting a study each time. Additionally, a subgroup analysis and meta-regression was conducted to detect the potential factors for heterogeneity. Statistical analysis was performed using the STATA version 15 (StataCorp, College Station, TX).

## Results

### Search Results

**Figure 1:**
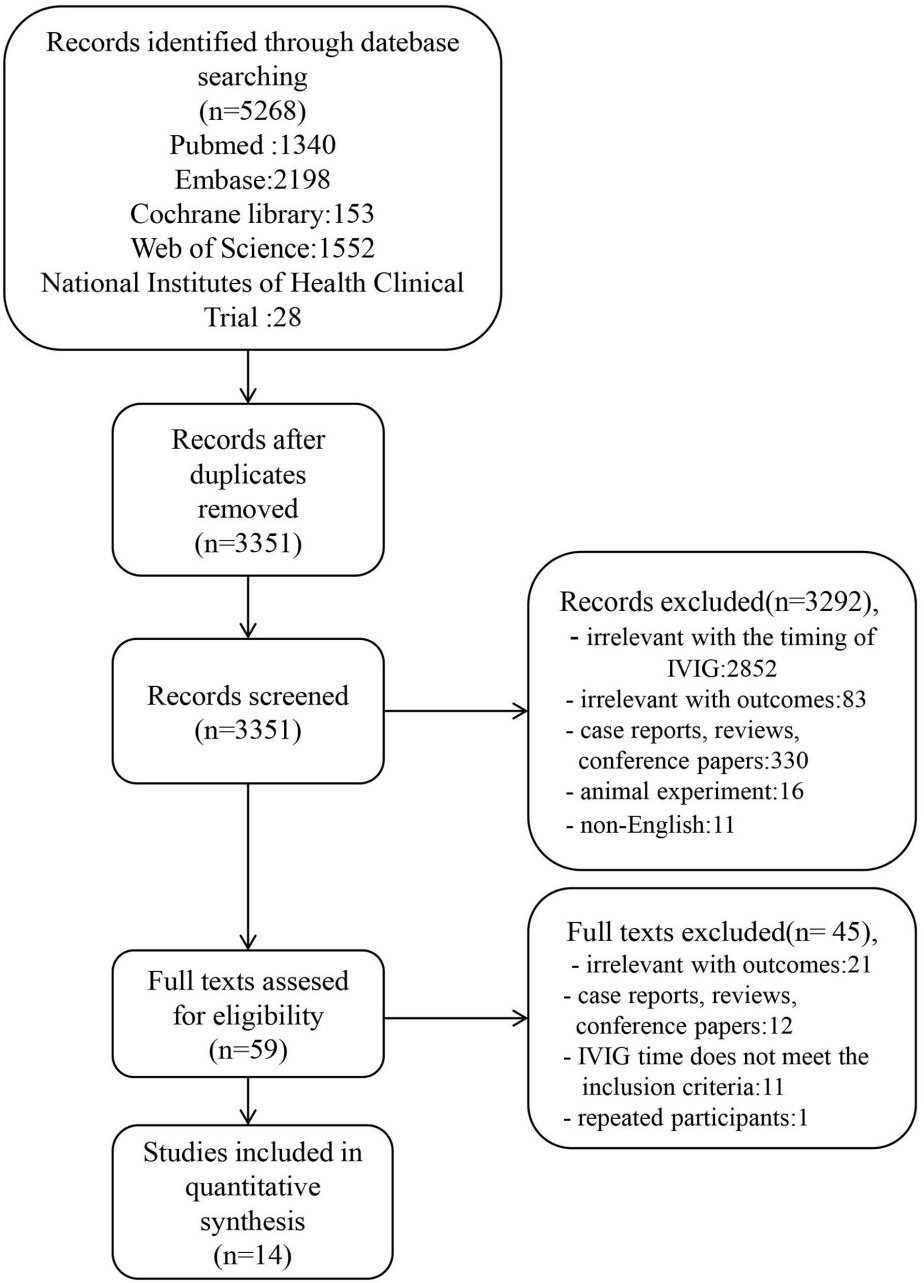
The flow chart of the included studies.

After searching for and removing the duplicates, a total of 3,351 titles and abstracts of relevant articles were checked. After the above screening, 59 articles were read in full and 15 articles met the inclusion criteria. Among them, the participants included in the study of Muta and Abrams overlapped (8, 16). Since the subjects included in Abrams had a larger time frame, the study of Muta was excluded. The final 14 studies were enrolled in this meta-analysis. The flow chart of the included studies is shown in Figure 1. The characteristics of the 14 enrolled studies involving a total of 70,396 patients are summarized in Table 1 (9, 16–28). The outcomes of the eight studies included the effects of early IVIG administration on the occurrence of CAL (17–21, 26–28), 10 studies involved IVIG unresponsiveness (9, 17–20, 22–25, 28), and CAA (9, 20) was investigated in two studies.

**Table 1:** Summary of the included studies for quantitative synthesis.

**References**	**Study design**	**Duration of following-up**	**Study duration**	**Study location**	**Year**	**Male(%)**	**Sample size in each group(time of IVIG), No**.	**Diagnostic criteria of CAL**	**Define of IVIG-resistant KD**	**Outcomes**	**NOS score(stars)**
Chen et al. (17)	Retrospectively study	NR	2008–2012	China	32 days to 11.7 years	1442 (62.6%)	< 5 d 289 5–10 d 1726 >10 d 157	Japanese criteria	NR	CAL IVIG Unresponsiveness,	5
Masanari et al. (18)	Cohort study	Over 1 month	2011–2012	Japan	IVIG < 5 d 783 days (mean) IVIG 5–7 d 977 days (mean) IVIG>7 d 1,337 days (mean)	IVIG < 5 d 41% IVIG 5–7 d 43% IVIG>7 d 40%	<5 d 6926 5–7 d 13295 >7 d 624	Japanese criteria	NR	CAL IVIG Unresponsiveness Additional IVIG therapy	6
Shiozawa et al. (19)	Retrospectively study	1 month	2006–2013	Japan	10–36.5 months	128 (64%)	IVIG < 5 d 100 IVIG = 5 d 100	Japanese criteria	Need for additional treatment because of persistent fever or relapsing fever associated with other KD symptoms after resolution of fever	CAL Additional IVIG therapy IVIG-resistance	8
Abrams et al. (16)	Retrospectively study	NR	1997–2004	Japan	<18 years	NR	<5 d 14134 5–10 d 34176	NR	NR	Additional IVIG, Giant aneurysms, All aneurysms, CAL, Major cardiac complications	6
Du et al. (20)	Cohort study	6 weeks	2000–2004	China	2 months–l3 year	680 (64.6%)	<5 d 108 5–9 d 763 ≥10 d 181	NR	Persistent fever (>38.5°C) lasted more than 48 h or recrudescent fever associated with KD symptoms after the first IVIG infusion	CAL IVIG Unresponsiveness, Aneurysm	6
Callinan et al. (21)	Retrospectively study	NR	2000–2009	America	<18 years	1106 (60.6%)	<5 d 433 ≥5 d 1273	NR	NR	CAL	6
Kobayashi (22)	Retrospectively study	30 days	2000-2006	Japan	1–119 months	315 (58%)	<5 d NR ≥5 d NR	Japanese criteria	Persistent fever lasted more than 24 hours or recrudescent fever associated with KD symptoms after an afebrile period.	IVIG Unresponsiveness,	6
Fu et al. (23)	Retrospectively study	NR	2002–2010	China	2 months−14 years	746 (63.4%)	<5 d NR 5–10 d NR	Z scores	Persistent or recurrent fever at any time 48 h to 2 weeks after initial IVIG treatment and with at least 1 of the standard diagnostic criteria of KD	IVIG Unresponsiveness,	5
Egami et al. (24)	Retrospectively study	1 month	1998–2004	Japan	≤ 6 months 34 7–60 months 258 ≥61 months 28	183 (57,2%)	<5 d NR ≥5 d NR	Japanese criteria	Responder as a patient who showed resolution of fever (<37.5°C) and a fall in CRP by more than 50% within 48 h after initial IVIG treatment	IVIG Unresponsiveness	6
Fong et al. (9)	Case–control study	After the diagnosis of KD, at weeks 2, 4, and 8 and yearly	1994–1999	Hong Kong	<5 d 31.8 months (mean) 5–10 d 24.2 months (mean)	50(61.7%)	<5 d 15 5–10 d 66	NR	NR	Persistent fever, Coronary aneurysm, Additional dosesof IVIG infused	8
Tremoulet et al. (25)	Retrospectively study	At the time of KD diagnosis and 2–4 weeks	1998-2006	AmerIca	2.3 years (mean)	NR	<5 d NR 5–10 d NR	NR	Persistent or recrudescent fever (T ≥ 100.4°F rectally or orally) at least 48 h but not longer than 7 days after completion of the first IVIG infusion	IVIG-resistance	6
Li et al. (26)	Retrospectively study	>8 weeks	2008–2012	China	3 months−16.3 years	321(60.9%)	<5 d 131 5–10 d 293 >10 d 103	Chinese criteria	Persistent fever that lasted more than 36 h or recrudescent fever associated with KD symptoms after the first IVIG infusion	CAL	6
Yong-Chao et al. (27)	Case–control study	NR	2012–2014	China	1 months−16.2 years	578 (64.6%)	<5 d 370 ≥5 d 525	NR	Persistent fever that lasted more than 36 h or recrudescent fever associated with KD symptoms after the first IVIG infusion	CAL	6
Hsieh et al. (28)	Retrospectively study	At the time of KD diagnosis and again at weeks 2, 4, and 8 after treatment and annually	1993-2003	Taiwan	2 months−7.8 years	100 (61.7%)	<5 d 16 ≥5 d 146	Japanese criteria	Fever persisted for _3 days after IVIG treatment	IVIG-nonresponsive, CAL,	9

*NOS, Newcastle-Ottawa Quality Assessment Scale; NR, no record; CAL, coronary artery lesions; IVIG, intravenous immunoglobulin; Z scores, body surface area adjusted z-scores*.

### Primary Outcomes

#### CAL

**Figure 2:**
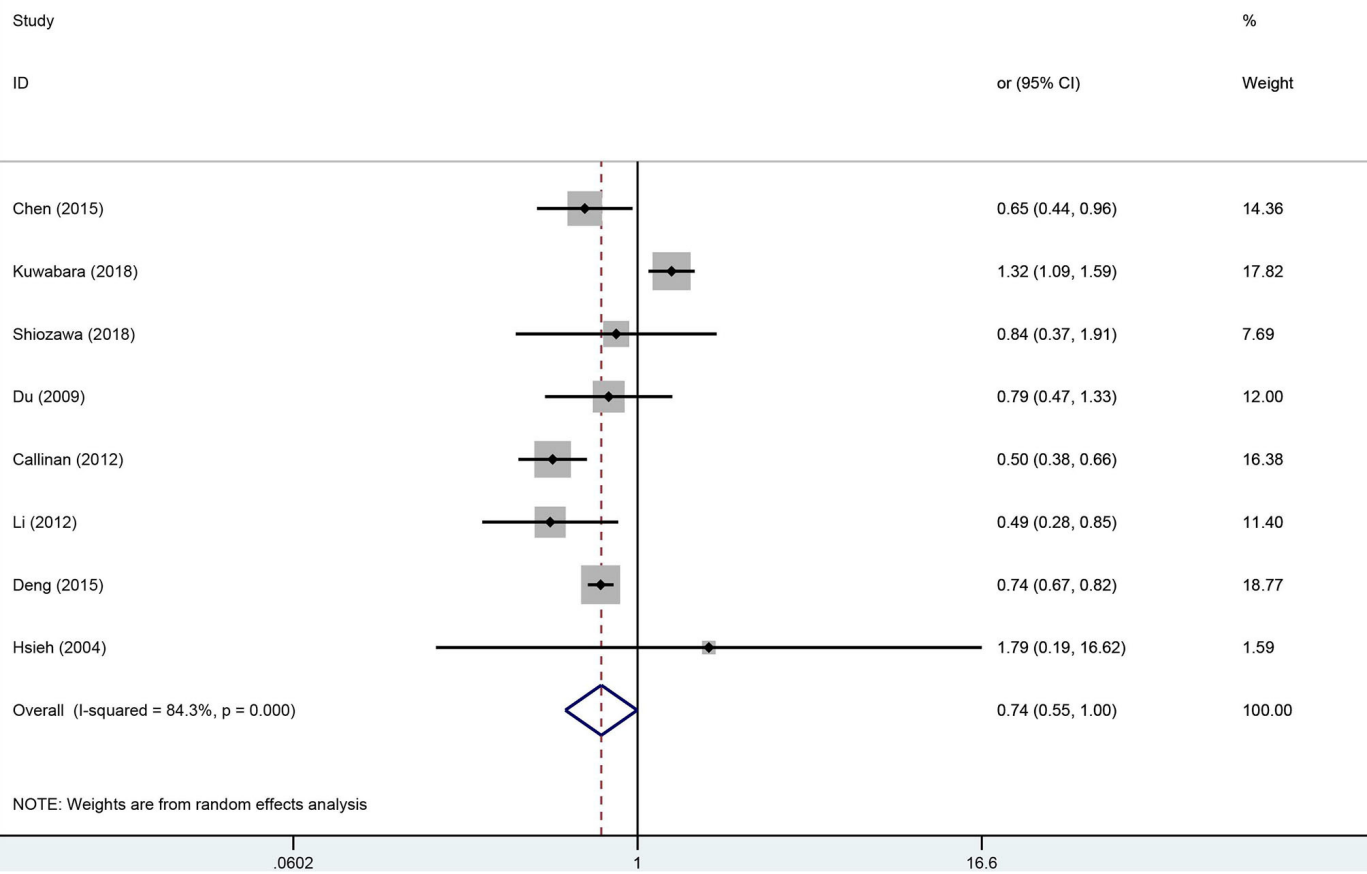
Pooled odds ratio for CAL development by the timing of IVIG therapy in KD (<5 days of disease onset vs. ≥5 days).

Meta-analysis for the pooled OR in the early treatment with IVIG on CAL is shown in Figure 2. With upper 95% CI = 1.0, there was no evidence supporting significant differences in the incidence of CAL between the early treatment with IVIG and 5 days after onset [OR 0.74; 95% CI (0.55, 1.00); *P* = 0.048]. However, significant heterogeneity was detected between the studies (*P* < 0.01; *I*^2^ = 84.3%). Begg's test (*P* = 0.266) and Egger's test (*P* = 0.263) showed no marked asymmetry.

**Figure 3:**
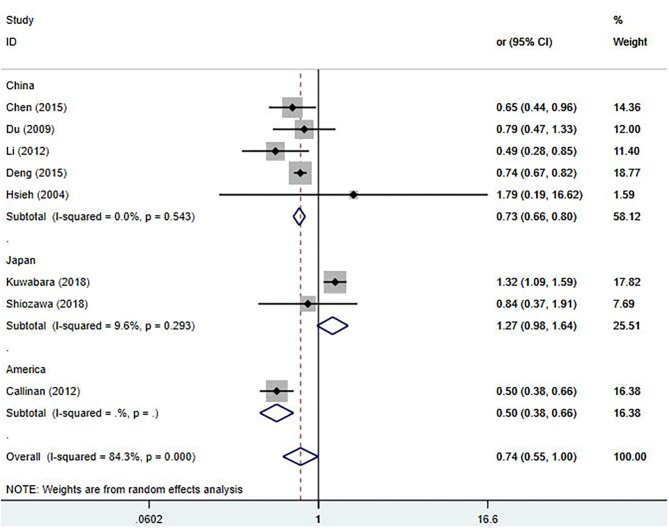
Pooled odds ratio for CAL development by the timing of IVIG therapy in KD (subgroup analysis basing on the study location).

For sensitivity analysis, after omitting a study each time, the overall result was still stable (Supplementary Figure 1). To find the potential sources of heterogeneity, we used the study location, CAL diagnostic criteria, and duration of follow-up as covariates for the meta-regression analysis (Supplementary Table 3). The meta-regression results showed that the study location influenced the pooled estimate (China, *P* = 0.003, America, *P* = 0.003). A subgroup analysis was performed based on the study locations (Figure 3), we observed a significant decrease in the heterogeneity. From the overall results by each region, the Chinese studies supported that early treatment with IVIG was a protective factor against the developing CAL [OR 0.73; 95% CI (0.66, 0.80); *P* < 0.001] with a low heterogeneity (*P* = 0.543; *I*^2^ = 0.0%); however, the Japanese studies failed to show significant differences in the incidence of CAL regardless of the timing of IVIG treatment [OR 1.27; 95% CI (0.98, 1.64); *P* = 0.074]. Callinan's study was the only American study enrolled in this meta-analysis [OR 0.50; 95% CI (0.38, 0.66); *P* < 0.001].

#### IVIG Unresponsiveness

**Figure 4:**
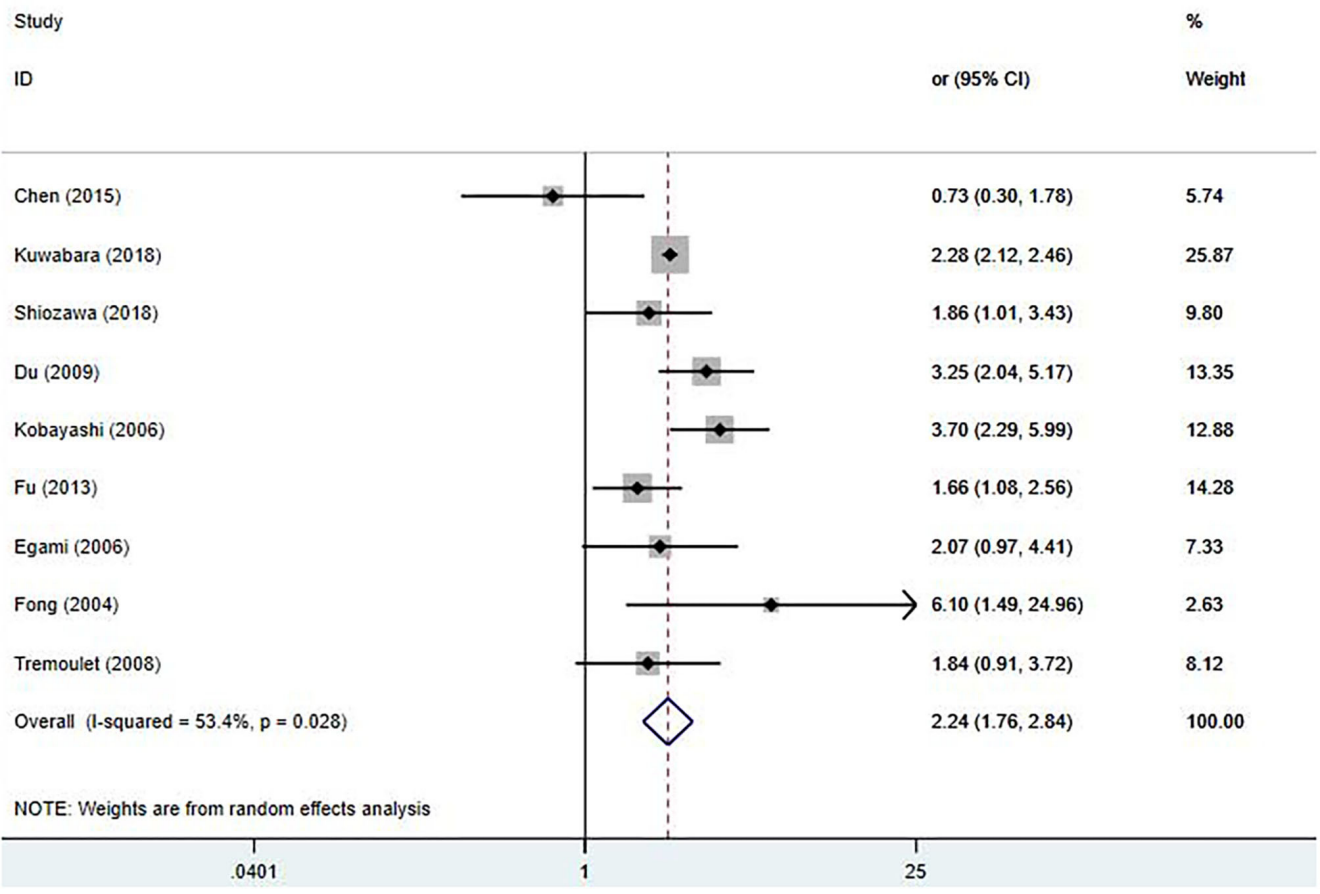
Pooled odds ratio for IVIG unresponsiveness by the timing of IVIG therapy in KD (<5 days of disease onset vs. ≥5 days).

Due to having a small sample size, Hsieh's study was excluded (28). Nine studies (9, 17–20, 22–25) involving IVIG unresponsiveness were included in this meta-analysis; results are shown in Figure 4. Early treatment with IVIG was a significant risk factor for IVIG unresponsiveness [OR 2.24; 95% CI (1.76, 2.84); *P* < 0.001]. However, a significant heterogeneity was observed (*P* = 0.028; *I*^2^ = 53.4%). Begg's test (*P* = 0.917) and Egger's test (*P* = 0.877) showed no marked asymmetry.

In the sensitivity analysis, the total findings did not change significantly (Supplementary Figure 2). It was noticed that Chen's study (17) reported an extremely low incidence of IVIG unresponsiveness (4.9%), which was far lower than the other studies (12.3–38.3%). After omitting this study, the heterogeneity significantly decreased [OR 2.37; 95% CI (1.95, 2.90); *P* < 0.001; *I*^2^ = 35.5%, *P* = 0.145]. A meta-regression analysis, including the definition of IVIG unresponsiveness and study location, was also performed. However, these factors had no significant effect on the homogeneity of the included studies (Supplementary Table 4).

### Secondary Outcome

#### CAA

**Figure 5:**
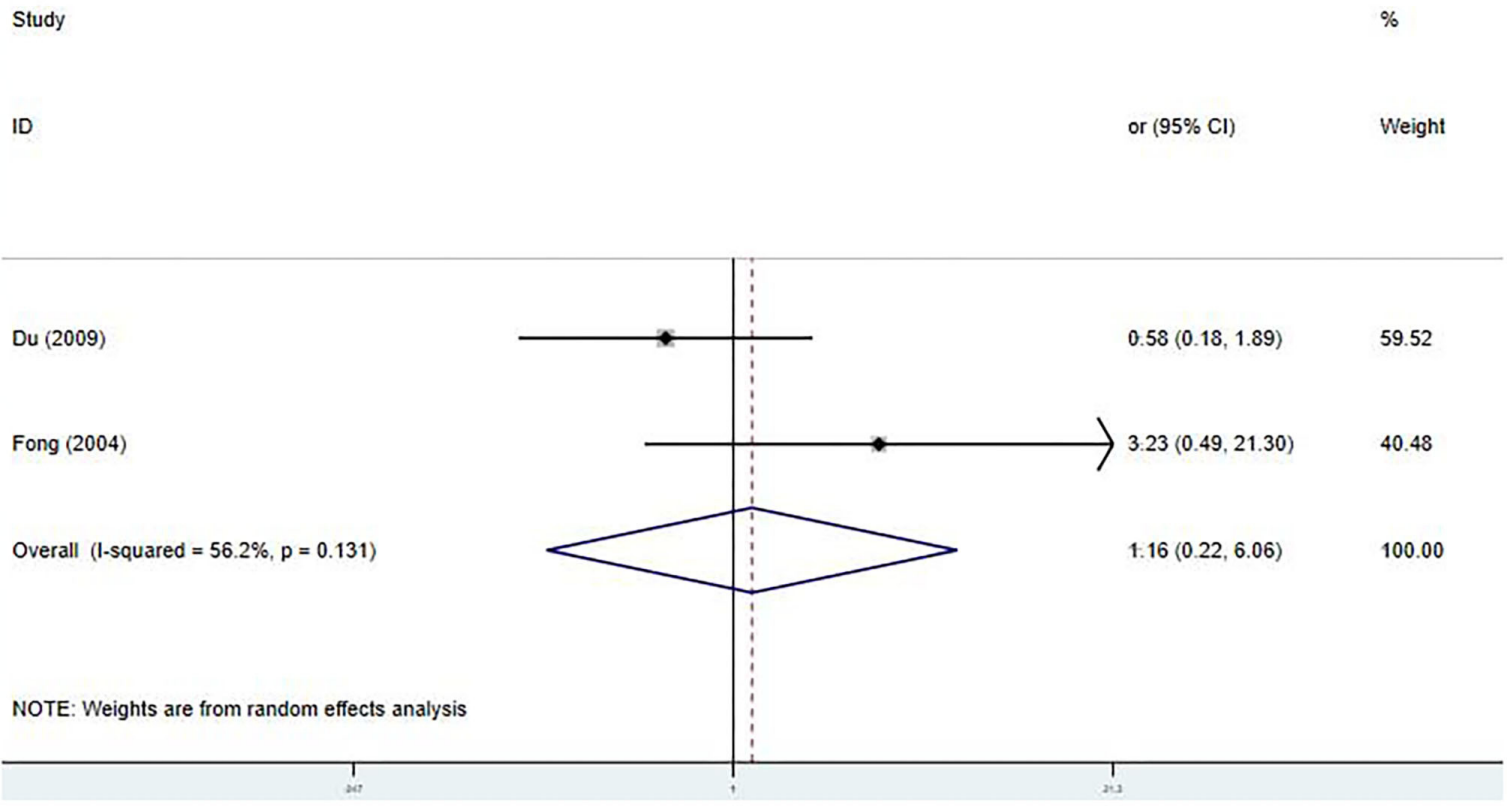
Pooled odds ratio for CAA development by the timing of IVIG therapy in KD (<5 days of disease onset vs. ≥5 days).

Only two studies were included in the meta-analysis of the relationship between the early IVIG treatment and the development of CAA (9, 20). We found no significant differences in the incidence and a significant heterogeneity between the studies [OR 1.16; 95% CI (0.22, 6.06); *P* = 0.858; *I*^2^ = 56.2%, *P* = 0.131] (Figure 5).

## Discussion

KD is characterized by a persistent fever in addition to five typical clinical manifestations (29) and can lead to systemic vasculitis, especially damaging the coronary arteries (30). The most effective treatment is known to be IVIG, which is recommended as a 2 g/kg single infusion 5–10 days after the onset of KD (4). Various studies have shown that the patients with a delayed IVIG treatment have a higher incidence of CAL and IVIG resistance (20, 31–33). However, current researches on the early use of IVIG is somewhat controversial.

It was reported that after the acute phase of the KD, 1,356 patients were followed-up for up to 15 years, the coronary artery events increased significantly in the patients with severe coronary dilatation. In patients with both a Z score ≥10 and an absolute dimension ≥8 mm, the incidence was as high as 48% (34). Therefore, CAL has been the focus for clinicians and researchers for a long time. Earlier literature reported that the incidence of CAL dropped from 25 to 4% after the conventional IVIG and aspirin treatment (35, 36). However, the incidence rates reported in recent years have risen significantly. In a nationwide survey on the epidemiology of KD in Japan (2011–2012), 9.3% of patients had one or more cardiac lesions (37). In 2012, Callinan reported a higher CAL incidence of 19% in California (21). Several studies have been conducted in order to reduce the occurrence of CAL, for example involving adjunctive corticosteroid therapy, different doses of aspirin, and tumor necrosis factor inhibitors in immunoglobulin-resistant KD (3, 38, 39). Unfortunately, they all failed to find convincing positive results.

This meta-analysis was performed to investigate the effects of IVIG therapy timing on the outcomes of KD. Overall, the early use of IVIG did not significantly reduce the incidence of CAL and CAA. However, the meta-regression analysis found that the study location had a significant influence on the outcomes. In the subgroup analysis, compared to the studies done in Japan, the studies conducted in China and the United States supported early IVIG treatment as a protective factor against the development of CAL. Considering the worldwide epidemiology, the incidence of KD and CAL in East Asia is significantly higher than that in Europe and the United States, it was reported that the KD incidence per 100,000 children were reported as 264.8 cases in Japan, 134.4 cases in Korea, 82.7 in Taiwan, 51.4 in Singapore, 21.9 in Canada, 18.1 in the United States, 15.2 in Ireland, and 9 in France (40, 41). In addition, a few scoring systems used to predict the IVIG resistance are highly sensitive and specific in the Japanese population but they have not been as useful in other areas (42, 43). Thus, the characteristics of KD are thought to vary between ethnicities, which may be the reason for the difference. However, considering the study design, the results must be interpreted with caution. Part of the studies we included were a comparison of the IVIG intervention within 5 days and after 5 days, including 10 days after the KD onset. When we meta-analyzed four studies comparing IVIG treatment within 5 days and within 5–10 days, there were no significant differences in the CAL outcomes (Supplementary Figure 3). In addition, the different definitions of CAL and the length of follow-up after KD onset may also influence the results.

Moreover, patient-related factors are speculated to be an important risk factor for CAL development, too. Patients undergoing the early IVIG treatment may be inclined to have more typical clinical manifestations and more intense inflammatory responses than patients with the conventional or delayed IVIG treatment, leading to an early introduction of IVIG treatment that could lead to CAL development. Thus, more well-designed randomized controlled study is important for a rigorous conclusion regarding the impact of IVIG timing on the development of CAL.

The 2003–2004, 2007–2008, and 2011–2012 Nationwide Surveys in Japan reported the incidence of IVIG unresponsiveness to be 26.4, 22.1, and 17%, respectively (8, 37, 44, 45). Tremoulet reported an incidence of IVIG unresponsiveness of up to 38.3% in San Diego in 2006 (25). In a previous meta-analysis, it was found that the patients who were IVIG-unresponsive had a 3.43 times higher risk of developing CAA (46). Therefore, by the early identification of patients with IVIG unresponsiveness and active administration of additional IVIG supplemented by other treatments, the incidence of CAL may be further reduced.

Several IVIG unresponsiveness scoring systems have been published. The main factors for IVIG unresponsiveness are the days of illness at initial treatment, neutrophil percentage and count, C-reactive protein (CRP), and age (23). We also found that early infusion of IVIG is a risk factor for IVIG unresponsiveness. However, patient–related factors have been speculated to be a cause of IVIG unresponsiveness in patients who received early IVIG treatment. In the Kobayashi scoring system established in Japan, Kobayashi found that the patients in the early IVIG treatment group had higher scores regardless of the IVIG treatment time. Therefore, it is speculated that the patients receiving IVIG at an early stage may have had a more severe form of KD (22). In 2018, Shiozawa et al. conducted a study with a better design to control the impact of patients' baseline characteristics. They divided the study subjects into two groups: one group was given IVIG within 5 days after the onset of KD while the other group who were diagnosed early were not treated with IVIG until day five. They, then, adjusted for known confounders using the propensity scores. A higher rate of treatment resistance still appeared in the early treatment group (19). It was suggested that the timing of IVIG treatment influenced the reactivity to treatment regardless of the severity of KD. Besides, It was reported that in patients with KD, inflammatory mediators, including plasma white blood cell count, absolute neutrophil count, and CRP peak within 5–10 days of KD onset, thus, the inappropriate timing of IVIG treatment may lead to an increased incidence of IVIG unresponsiveness (47). However, owing to the potential confounders between groups, subsequent studies are still needed to focus on controlling for differences in patients' baseline characteristics in order to obtain reliable results.

## Study Limitations

Our study has several limitations. First, all included studies were non-randomized retrospective studies. Second, there were slight differences in the CAL diagnostic criteria, IVIG unresponsiveness criteria, and the time of follow-up. Third, the grouping of IVIG infusion time points in the different literatures have some differences, which limits the number of included studies. Moreover, a few studies had slightly smaller sample sizes, such as studies of Hsieh et al. and Fong et al. Thus, more randomized controlled studies with careful controlling of the confounders between groups following the standard treatment and follow-up procedures are needed to identify better time points for IVIG therapy.

## Conclusion

In conclusion, early treatment with IVIG can lead to an increased risk of IVIG unresponsiveness. In contrast to the studies performed in Japan that found no significant difference in coronary outcomes, the studies conducted in China and the United States showed a reduced risk in the occurrence of CAL with an early IVIG treatment. At present, the evidence does not support the treatment with IVIG in the early stage of the onset of KD. But, early IVIG treatment could be a protective factor against the development of CAL, which needs to be further clarified.

## Data Availability Statement

All datasets generated for this study are included in the article/Supplementary Material.

## Informed Consent

This systematic review and meta-analysis is based on a collection of data retrieved from studies that have already been published. We did not collect individual patient data and did not have direct contact with any of the included patients.

## Author Contributions

FY, HZ, and FZ designed and conceived the experiments. FY and HZ performed the experiments. FY, RX, and XC analyzed the data. XC and YC contributed the reagents, materials, and analysis tools. FY, RX, and FZ wrote the manuscript. All authors contributed to the article and approved the submitted version.

## Conflict of Interest

The authors declare that the research was conducted in the absence of any commercial or financial relationships that could be construed as a potential conflict of interest.

## Supplementary Material

Supplementary Figure 1 Sensitivity analysis of the included studies for the incidence analysis of CAL.

Supplementary Figure 2 Sensitivity analysis of the included studies for the incidence analysis of IVIG unresponsiveness.

Supplementary Figure 3 Pooled odds ratio for CAL development by the timing of IVIG therapy in KD (<5 days of disease onset vs. 5–10 days).

Supplementary Table 1 The NEWCASTLE-OTTAWA SCALE for case control studies.

Supplementary Table 2 The NEWCASTLE-OTTAWA SCALE for cohort studies.

Supplementary Table 3 Meta-regression analysis of the included studies for the primary outcome (CAL development).

Supplementary Table 4 Meta-regression analysis of the included studies for the primary outcome (IVIG unresponsiveness).

Supplementary Checklist 1 PRISMA checklist.
