# Supplementary figures and images for: Effect of Early Intravenous Immunoglobulin Therapy in Kawasaki Disease: A Systematic Review and Meta-Analysis
Source: Front Pediatr. 2020 Nov 20;8:593435. doi: 10.3389/fped.2020.593435 (PMC7715029; doi:10.3389/fped.2020.593435)

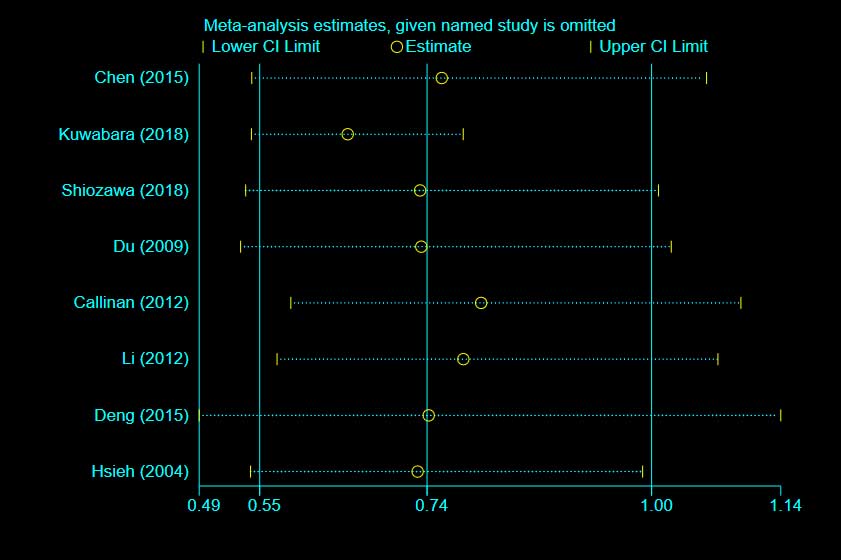

Supplement: Supplementary Figure 1 — Sensitivity analysis of the included studies for the incidence analysis of CAL. [file Image_1.JPEG]

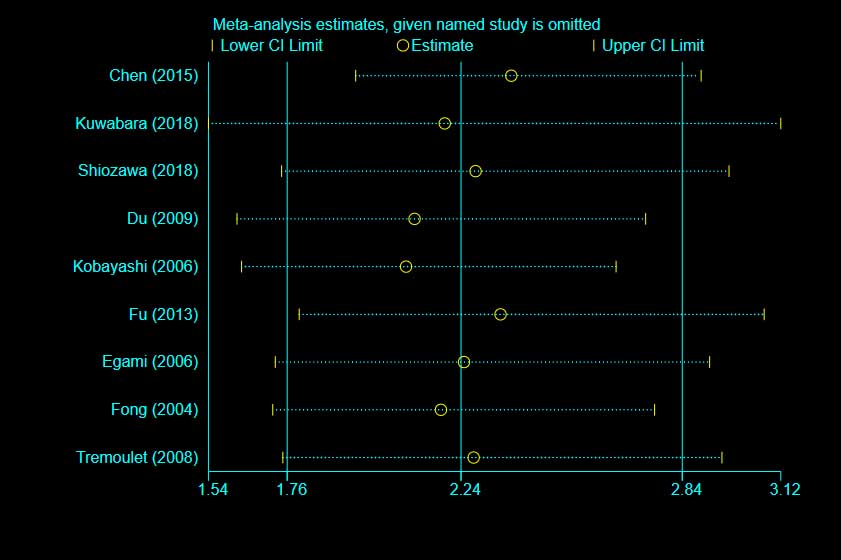

Supplement: Supplementary Figure 2 — Sensitivity analysis of the included studies for the incidence analysis of IVIG unresponsiveness. [file Image_2.JPEG]

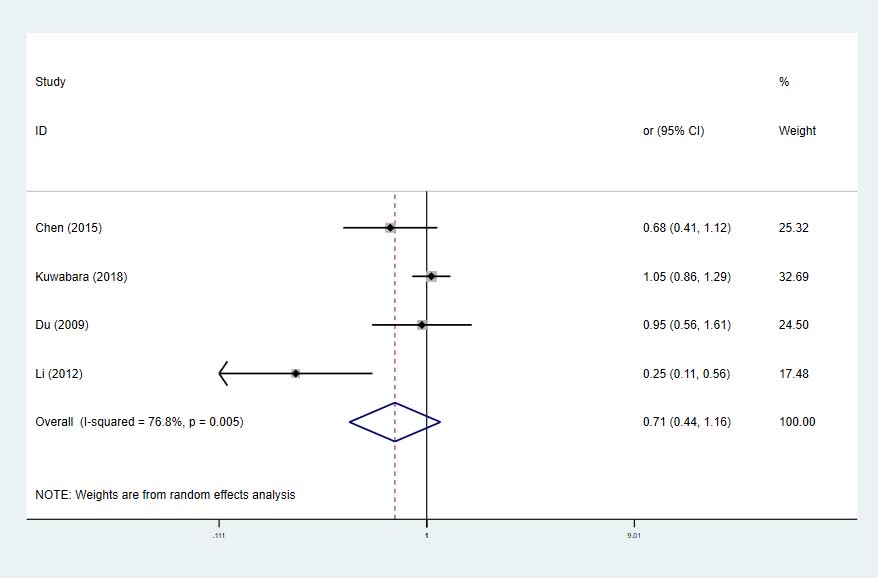

Supplement: Supplementary Figure 3 — Pooled odds ratio for CAL development by the timing of IVIG therapy in KD (<5 days of disease onset vs. 5–10 days). [file Image_3.JPEG]
