# Supplementary material for: Effect of Early Intravenous Immunoglobulin Therapy in Kawasaki Disease: A Systematic Review and Meta-Analysis
Source: Front Pediatr. 2020 Nov 20;8:593435. doi: 10.3389/fped.2020.593435 (PMC7715029; doi:10.3389/fped.2020.593435)
Supplement: Supplementary Table 1 — The NEWCASTLE-OTTAWA SCALE for case control studies. [file Table_1.docx]

| First  Author, Year | selection | | | | Comparability | Exposure | | | Total score |
| --- | --- | --- | --- | --- | --- | --- | --- | --- | --- |
|  | Is the case definition adequate | Representativeness of the cases | Selection of Controls | Definition of Controls |  | Ascertainment of exposure | Same method  of ascertainment for cases and controls | Non-Response rate |  |
| Chen, 2015 | 1 | 1 | 0 | 1 | 0 | 1 | 1 | 0 | 5 |
| Shiozawa, 2018 | 1 | 1 | 0 | 1 | 2 | 1 | 1 | 1 | 8 |
| Abrams, 2017 | 1 | 1 | 1 | 1 | 0 | 1 | 1 | 0 | 6 |
| Callinan, 2012 | 1 | 1 | 1 | 1 | 0 | 1 | 1 | 0 | 6 |
| Kobayashi, 2006 | 1 | 1 | 1 | 1 | 0 | 1 | 1 | 0 | 6 |
| Fu, 2013 | 1 | 1 | 0 | 1 | 0 | 1 | 1 | 0 | 5 |
| Egami, 2006 | 1 | 1 | 0 | 1 | 0 | 1 | 1 | 1 | 6 |
| Tremoulet, 2008 | 1 | 1 | 1 | 1 | 0 | 1 | 1 | 0 | 6 |
| Li Y, 2012 | 1 | 1 | 1 | 1 | 0 | 1 | 1 | 0 | 6 |
| Hsieh, 2004 | 1 | 1 | 1 | 1 | 2 | 1 | 1 | 1 | 9 |
|  |  |  |  |  |  |  |  |  |  |
|  |  |  |  |  |  |  |  |  |  |
|  |  |  |  |  |  |  |  |  |  |
|  |  |  |  |  |  |  |  |  |  |
|  |  |  |  |  |  |  |  |  |  |
|  |  |  |  |  |  |  |  |  |  |
|  |  |  |  |  |  |  |  |  |  |

**Supporting Table1. The NEWCASTLE-OTTAWA SCALE for case control studies.**
