# Supplementary material for: Effect of Early Intravenous Immunoglobulin Therapy in Kawasaki Disease: A Systematic Review and Meta-Analysis
Source: Front Pediatr. 2020 Nov 20;8:593435. doi: 10.3389/fped.2020.593435 (PMC7715029; doi:10.3389/fped.2020.593435)
Supplement: Supplementary Table 2 — The NEWCASTLE-OTTAWA SCALE for cohort studies. [file Table_2.docx]

| First Author, Year | selection | | | | Comparability | Outcome | | | Total score |
| --- | --- | --- | --- | --- | --- | --- | --- | --- | --- |
|  | Representativeness of the exposed cohort | Selection of the non-exposed cohort | Ascertainment of exposure | Demonstration that outcome of interest was not present at start of study |  | Assessment of outcome | Was follow  -up long enough for outcomes to occur | Adequacy of follow up of cohorts |  |
| Kuwabara, 2018 | 1 | 1 | 1 | 0 | 0 | 1 | 1 | 1 | 6 |
| Du, 2009 | 1 | 1 | 1 | 0 | 0 | 1 | 1 | 1 | 6 |
| Fong, 2004 | 1 | 1 | 1 | 0 | 2 | 1 | 1 | 1 | 8 |
| Deng，2015 | 1 | 1 | 1 | 0 | 0 | 1 | 1 | 1 | 6 |

**Supporting Table 2. The NEWCASTLE-OTTAWA SCALE for cohort studies.**
