# Supplementary material for: Effect of Early Intravenous Immunoglobulin Therapy in Kawasaki Disease: A Systematic Review and Meta-Analysis
Source: Front Pediatr. 2020 Nov 20;8:593435. doi: 10.3389/fped.2020.593435 (PMC7715029; doi:10.3389/fped.2020.593435)
Supplement: Supplementary Table 3 — Meta-regression analysis of the included studies for the primary outcome (CAL development). [file Table_3.docx]

| Study location | exp(b) | t | P>\|t\| | 95%CI | |
| --- | --- | --- | --- | --- | --- |
| America | .38749 | -5.55 | 0.003 | .2497597 | .6011719 |
| China | .5638014 | -5.39 | 0.003 | .4289168 | .7411041 |
| _cons | 1.290356 | 2.72 | 0.042 | 1.013726 | 1.642473 |

Freq. Percent Cum.

America | 1 12.50 12.50

China | 5 62.50 75.00

Japan | 2 25.00 100.00

***a. Meta-regression analysis of*** ***study location.***

| diagnostic criteria of CAL | exp(b) | t | P>\|t\| | 95%CI | |
| --- | --- | --- | --- | --- | --- |
| China | .7473243 | -0.66 | 0.541 | .2387439 | 2.3393 |
| Japan | 1.477818 | 1.41 | 0.219 | .723479 | 3.018673 |
| _cons | .6556725 | -2.24 | 0.075 | .4043044 | 1.063324 |

Freq. Percent Cum.

China | 1 12.50 12.50

Japan | 4 50.00 62.50

No record | 3 37.50 100.00

***b. Meta-regression analysis of CAL diagnostic criteria***

|  | exp(b) | t | P>\|t\| | 95%CI | |
| --- | --- | --- | --- | --- | --- |
| Duration of following-up | 1.385836 | 1.20 | 0.276 | .711939 | 2.697621 |

Freq

Record | 5

No record | 3

***c. Meta-regression analysis of duration of follow-up***

**Supporting Table 3. Meta-regression analysis of primary outcome (** CAL development)**).**
