# Supplementary material for: Effect of Early Intravenous Immunoglobulin Therapy in Kawasaki Disease: A Systematic Review and Meta-Analysis
Source: Front Pediatr. 2020 Nov 20;8:593435. doi: 10.3389/fped.2020.593435 (PMC7715029; doi:10.3389/fped.2020.593435)
Supplement: Supplementary Table 4 — Meta-regression analysis of the included studies for the primary outcome (IVIG unresponsiveness). [file Table_4.docx]

|  | exp(b) | t | P>\|t\| | 95%CI | |
| --- | --- | --- | --- | --- | --- |
| Define of IVIG- unresponsiveness | 1.187707 | 0.48 | 0.646 | .5081655 | 2.775963 |

Freq

Record | 6

No record| 3

***a. Meta-regression analysis of define of IVIG- unresponsiveness.***

| Study location | exp(b) | t | P>\|t\| | 95%CI | |
| --- | --- | --- | --- | --- | --- |
| China | 1.107432 | 0.16 | 0.880 | .2266718 | 5.410494 |
| japan | 1.318625 | 0.44 | 0.677 | .2810962 | 6.185686 |
| _cons | 1.84 | 1.05 | 0.333 | .445426 | 7.600814 |

Freq. Percent Cum.

America | 1 11.11 11.11

China | 4 44.44 55.56

Japan | 4 44.44 100.00

***b. Meta-regression analysis of study location.***

**Supporting Table 4. Meta-regression analysis of primary outcome (IVIG unresponsiveness).**
